# Efficacy and Safety of Direct Acting Antivirals in Kidney Transplant Recipients with Chronic Hepatitis C Virus Infection

**DOI:** 10.1371/journal.pone.0158431

**Published:** 2016-07-14

**Authors:** Ming V. Lin, Meghan E. Sise, Martha Pavlakis, Beth M. Amundsen, Donald Chute, Anna E. Rutherford, Raymond T. Chung, Michael P. Curry, Jasmine M. Hanifi, Steve Gabardi, Anil Chandraker, Eliot C. Heher, Nahel Elias, Leonardo V. Riella

**Affiliations:** 1 Division of Gastroenterology, Hepatology and Endoscopy, Brigham and Women’s Hospital, Harvard Medical School, Boston, MA, United States of America; 2 Division of Nephrology, Massachusetts General Hospital, Harvard Medical School, Boston, MA, United States of America; 3 Division of Nephrology, Beth Israel Deaconess Medical Center, Harvard Medical School, Boston, MA, United States of America; 4 Division of Transplant Surgery, Massachusetts General Hospital, Harvard Medical School, Boston, MA, United States of America; 5 Liver Center, Division of Gastroenterology, Massachusetts General Hospital, Harvard Medical School, Boston, MA, United States of America; 6 Division of Gastroenterology and Hepatology, Beth Israel Deaconess Medical Center, Harvard Medical School, Boston, MA, United States of America; 7 Department of Internal Medicine, Brigham and Women’s Hospital, Harvard Medical School, Boston, MA, United States of America; 8 Transplantation Research Center, Renal Division, Brigham & Women's Hospital, Harvard Medical School, Boston, MA, United States of America; National Taiwan University Hospital, TAIWAN

## Abstract

The prevalence of Hepatitis C Virus (HCV) infection is significantly higher in patients with end-stage renal disease compared to the general population and poses important clinical challenges in patients who undergo kidney transplantation. Historically, interferon-based treatment options have been limited by low rates of efficacy and significant side effects, including risk of precipitating rejection. Limited data exist on the use of all-oral, interferon-free direct-acting antiviral (DAA) therapies in kidney transplant recipients. In this study, we performed a retrospective chart review with prospective clinical follow-up of post-kidney transplant patients treated with DAA therapies at three major hospitals in Boston, MA. A total of 24 kidney recipients with HCV infection received all-oral DAA therapy post-transplant. Patients were predominantly male (79%) with a median age of 60 years (range 34–70 years), median creatinine of 1.2 mg/dL (0.66–1.76), and 42% had advanced fibrosis or cirrhosis. The majority had HCV genotype 1a infection (58%). All patients received full-dose sofosbuvir; it was paired with simeprevir (9 patients without and 3 patients with ribavirin), ledipasvir (7 patients without and 1 patient with ribavirin) or ribavirin alone (4 patients). The overall sustained virologic response (SVR12) was 91% (21 out of 23 patients). One patient achieved SVR4 but demised prior to SVR12 check point due to treatment unrelated cause. Two treatment failures were successfully retreated with alternative DAA regimens and achieved SVR. Both initials failures occurred in patients with advanced fibrosis or cirrhosis, with genotype 1a infection, and prior HCV treatment failure. Adverse events were reported in 11 patients (46%) and were managed clinically without discontinuation of therapy. Calcineurin inhibitor trough levels did not significantly change during therapy. In this multi-center series of patients, all-oral DAA therapy appears to be safe and effective in post-kidney transplant patients with chronic HCV infection.

## Introduction

Hepatitis C virus (HCV) infection affects more than 200 million people worldwide and is highly prevalent in patients with end-stage renal disease, leading to significant challenges in kidney transplant recipients. In developed countries, approximately 1.8% to 8% of kidney transplant recipients are infected with HCV.[1,2]

HCV infection has a negative impact on both patient and graft survival in kidney transplant recipients compared to their HCV-negative counterparts.[3,4] Immunosuppression has a permissive effect on viral replication and can lead to progression of liver disease or reactivation of HCV infection and acute hepatitis after kidney transplantation.[5–10] Over the long term, HCV-infected patients are more susceptible to developing cirrhosis and hepatocellular carcinoma (HCC) after transplantation.[5] A recent meta-analysis demonstrated an adjusted relative risk for all-cause mortality of 1.85 and all-cause graft loss of 1.76 in kidney transplant recipients with HCV infection.[11] Liver disease, cardiovascular disease and infectious complications accounted for the top three causes of death.[11] However, despite these risks, it is still recommended that HCV-infected patients undergo kidney transplantation, as their mortality has been clearly demonstrated to improve after transplant compared to remaining on hemodialysis.[12,13]

Treatment of HCV post-transplant may mitigate some of the above risks; however, very few post-transplant patients received curative HCV treatment. Interferon and ribavirin have been the mainstay of therapy for HCV infection for the last 30 years. However, interferon-containing HCV regimens are rarely used in the kidney transplant population because they are associated with low efficacy (18%-34%), poor tolerability (drop-out rate of 25% to 32%) and high risk of irreversible interferon-induced graft rejection (12.5% to 51%).[14–17] Because of these risks, the latest Kidney Disease: Improving Global Outcomes (KDIGO) recommendations suggest treating HCV prior to kidney transplantation and only recommend use of interferon and ribavirin in the post-transplant period in the case of fibrosing cholestatic hepatitis or life-threatening vasculitis.[18]

**Fig 1:**
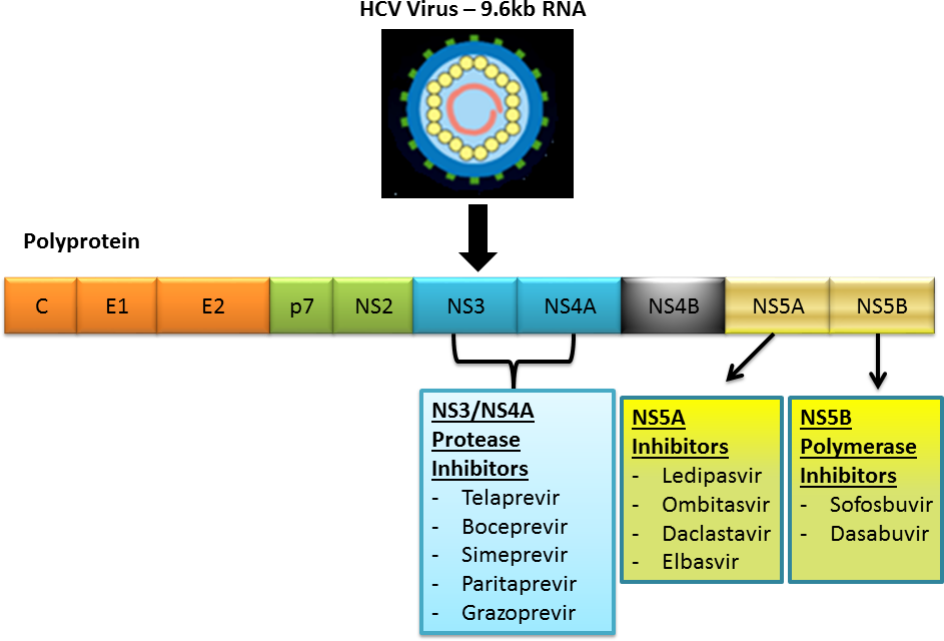
Hepatitis C Direct-Acting Antiviral. FDA approved direct-acting antiviral treatment for hepatitis C. HCV RNA is translated into a long polyprotein which consists of three structural proteins and seven non-structural (NS) proteins. The NS3/4A protease cleaves the downstream NS proteins into individual subunits. The major DAA classes consist of NS3/4A protease inhibitors, NS5A replication complex inhibitors and NS5B polymerase inhibitors.

Significant progress has been made in the development of oral HCV agents that target and directly inhibit different HCV viral proteins. The current generation of all-oral direct acting antiviral (DAA) therapies began with the approval of sofosbuvir, a nucleotide NS5B polymerase inhibitor in December 2013. There are now three classes of currently approved DAA agents targeting three viral proteins: NS5B polymerase, NS5A protein, and NS3/4A protease, shown in Fig 1. The efficacy of these oral agents used with ribavirin or in combination with one another yields a sustained virologic response at 12 weeks (SVR12) of greater than 90% among patients who are treatment naive.[19] However, the majority of the initial clinical trials for the DAAs have excluded kidney transplant recipients or patients with chronic kidney disease with estimated glomerular filtration rate less than 30ml/min, including those on hemodialysis. We sought to investigate the efficacy, tolerability, safety and viral kinetics of interferon-free DAA combination therapies in kidney transplant recipients with chronic HCV infection.

## Materials and Methods

### Study design and patients

This was a retrospective analysis with prospective follow-up of post-kidney transplant patients with chronic HCV treated with DAAs in three academic centers in Boston, Massachusetts: Beth Israel Deaconess Medical Center, Brigham and Women’s Hospital, and Massachusetts General Hospital. We identified patients by provider referral and through a search of the medical record system using the research patient data registry (RPDR) at Partners Healthcare. The RPDR database contained all patients’ records at Partners Healthcare System. We also cross checked these patients with the kidney transplant registry associated with each institution whom have underwent HCV treatment with the new DAAs. We included any adult patient aged 18 years and older diagnosed with chronic HCV infection, who had previously received a kidney transplant, which is functioning, and took at least one dose of a DAA therapy between 12/31/2013 and 08/31/2015. Patients were included irrespective of their liver fibrosis stage, genotype, or prior HCV treatment status. Sofosbuvir was prescribed at 400mg administered orally once daily; simeprevir was prescribed at 150mg administered orally once daily; ledipasvir was prescribed at 90mg administered orally once daily in co-formulation with sofosbuvir 400mg; ribavirin was prescribed according to body weight (1000mg daily in patients who were <75kg and 1200mg daily in patients who were > 75kg). Patients were followed prospectively. Demographic, clinical, virological and laboratory data were collected. Demographic data and clinical data were obtained at the time of enrollment. Virological and clinical data were collected at baseline, treatment weeks 2, 4, 8 and 12, and post-treatment week 12. Serum HCV RNA was measured using the COBAS® TaqMan® HCV Quantitative Test, v1.0, which has a lower limit of quantification of 43 IU per milliliter. HCV genotype and subtype were determined using VERSANT HCV LiPA 2.0. IL28B and Q80K polymorphisms were not included in the data analysis, since these were not routinely tested in our centers for patients being considered for this combination therapy. The study was approved by the institutional review board of the Harvard Catalyst and Partners Health Care System. The need for informed consent was waived.

### Clinical definitions

The presence of advanced fibrosis or cirrhosis was defined by one of three criteria: a liver-biopsy specimen showing evidence of advanced fibrosis (Ishak score 4) or cirrhosis (Ishak score 5–6); a FibroSure fibrosis score of 0.58–0.74 indicating advanced fibrosis and > 0.74 indicting cirrhosis; a FibroScan score of 10–12.5 kPa for advanced fibrosis and >12.5 kPa for cirrhosis. Patients were classified as treatment naïve if they had no prior therapy for their HCV infection and treatment experienced if they had received at least one dose of ribavirin, interferon, or a first generation protease inhibitor. Virologic non-response was defined as HCV RNA above the limit of detection throughout treatment. Virologic relapse was defined as HCV RNA below the limit of detection (<43 IU/mL) during treatment but which became detectable after cessation of treatment. Sustained virologic response was defined as undetectable serum HCV RNA at 12 weeks post-treatment (SVR 12). Estimated glomerular filtration rate (eGFR) was calculated using the CKD-EPI equation.[20]

### Outcomes

The primary efficacy endpoint was the rate of SVR12 after completion of therapy. This was assessed in the overall population and in the sub-groups stratified by prior treatment status, fibrosis stage and type of transplant, multiple organ (liver/kidney/pancreas), dual organ (liver/kidney or liver/pancreas) versus single organ (kidney) status. The safety end points included the rate of adverse events and any significant changes or interactions between the therapy and immunosuppressive agents in the post-transplant population.

### Follow-up and safety assessment

Patients were followed during treatment with scheduled clinic appointments with the HCV treating provider and kidney transplant nephrologist. Additional follow-up for 12 weeks after the completion of antiviral treatment was performed to evaluate the SVR 12. Routine laboratory testing were done at 4 weekly intervals and at 4 and 12 weeks after treatment completion, and these included complete blood counts, serum electrolytes, renal function panel, liver function tests, HCV RNA by the polymerase chain reaction technique and level of immunosuppressives (calcineurin inhibitor trough level–tacrolimus or cyclosporine). Compliance with antiviral therapy and adverse events were monitored through the treatment duration by the treating provider during scheduled clinic visits. Adverse events were determined by chart review; all clinical notes and laboratory results from the time of treatment initiation until four weeks after treatment completion were reviewed to determine adverse effects. Clinical outcomes including laboratory findings and kidney biopsy findings were determined by chart review for up to 24 weeks of follow-up.

### Statistical analysis

Baseline characteristics of all patients treated with DAA therapy were described using count and percent or median and range. Baseline and follow-up values were compared using paired samples t-test. Ninety-five percent confidence intervals were calculated using the exact formula. Signs and symptoms experienced during DAA therapy use were determined from chart review and the number of patients experiencing each potential adverse effect is tabulated. The data were analyzed with Statistix version 9.0 (Statistix, Tallahassee, Florida).

## Results

### Baseline patient characteristics

Twenty-four kidney transplant recipients with chronic HCV infection received DAA during the study period. Baseline patient demographic and clinical characteristics are summarized in Table 1. The majority of patients were white (48%), male (79%), and the median age was 60 years (range 34–70 years). The median time after kidney transplant to initiation of HCV therapy was 8 years (range from 2 months to 41 years). Sixteen patients received kidney transplant alone (67%), six had received liver and kidney transplants, one patient had received kidney and pancreas transplants, and one patient had received kidney, liver and pancreas. Nine patients (38%) had received two or more kidney transplants. The majority, 21 cases (88%), of patients had documented HCV infection prior to transplantation, though there were two cases (8%) that acquired HCV after receiving kidney transplantation (the source of infection was unknown). Seven patients (39%) received kidneys from HCV positive donors. The median creatinine at HCV treatment initiation was 1.21 mg/dL (range 0.66 to 1.76 mL/min/1.73m^2^) and the median eGFR was 71.9 mL/min/1.73m^2^ (range 47 to 96 mL/min/1.73m^2^).

**Table 1:** Demographic and Clinical Characteristics at Baseline.

Characteristics		Overall subjects (n = 24)*
Recipient age, median & range (years)		60; 34–70
Gender	Male	19 (79%)
	Female	5 (21%)
Race	Caucasian	11 (46%)
	African American	6 (25%)
	Hispanic	4 (17%)
	Asian	2 (8%)
	Unavailable	1 (4%)
BMI, median & range		25; 21.5–45.5
Time spent on dialysis, median, range (months)		24; 0–228
Duration from most recent (or last) kidney transplant to HCV treatment, median, range (months)		96; 2 to 492
Type of organ transplants	Kidney only	16 (67%)
	Kidney and liver	6 (25%)
	Kidney and pancreas	1 (4%)
	Kidney, liver and pancreas	1 (4%)
Number of kidney transplants	One	15 (62%)
	Two	7(30%)
	Three	2 (8%)
HCV status prior to transplant	Positive	22 (92%)
	Negative	2 (8%)
HCV status of donor organ	Positive	7 (29%)
	Negative	11 (46%)
	Not available	6 (25%)
HCV genotype	1a	14 (58%)
	1b	4 (17%)
	1 (non-subtypable)	3 (12.5%)
	2	3 (12.53%)
History of previous treatment	Treatment Naive	12 (50%)
	Prior treatment failure	12 (50%)
Metavir fibrosis stage	F0-F2	14 (58%)
	F3-F4	10 (42%)
Hepatic decompensation		5
HCV Viral Load, IU/mL, median, range		1,922,552; 1060–22,600,000
HCV RNA > 800,000 IU/mL		16 (67%)
Serum creatinine at treatment initiation, median, mg/dL		1.21; 0.66–1.76
eGFR, median, mL/min/1.73m^2^		71.9
eGFR, range, mL/min/1.73m^2^		47–96
Baseline Immunosuppression regimen**	Tacrolimus-based	19
	Cyclosporine-based	3
	Sirolimus-based	1

Abbreviations: BMI = body mass index, HCV = hepatitis C virus, eGFR = estimated glomerular filtration rate.

* Data presented are count and percentage or median and range where appropriate.

** The immunosuppression regimen is divided into tacrolimus-, cyclosporine- and sirolimus- based regimens, and these are mostly used in combination with other immunosuppressants such as prednisone, mycophenolate and/or azathioprine.

### Baseline viral characteristics

Baseline genotypes are shown in Table 1; the majority had genotype 1a infection (58%). Ten patients (42%) had advanced fibrosis or cirrhosis. Hepatic decompensation had occurred in five patients prior to treatment initiation—four of the cirrhotic patients and one non-cirrhotic patient who had fibrosing cholestatic hepatitis. All the cirrhotic patients were Child-Pugh class A at time of treatment initiation. High viral load (defined as HCV RNA greater than 800,000 IU/mL) was noted in 67% of the patient population.

### Antiviral regimens

**Fig 2:**
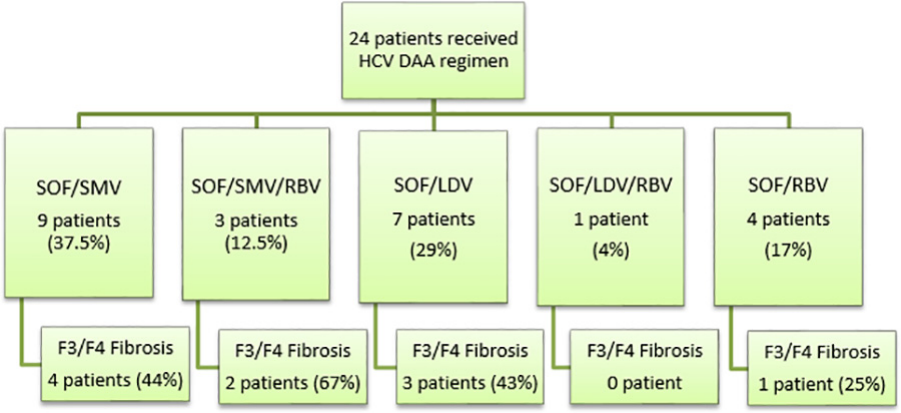
Study Population. HCV DAA regimen stratification of the study population. A flow chart of the study population, stratifying by the type/combination of DAA regimens patients received. Abbreviations SOF: sofosbuvir; SMV: simeprevir; LDV: ledipasvir; RBV: ribavirin

Three different oral regimens were prescribed: 1) Sofosbuvir and simeprevir, with or without ribavirin. 2) Sofosbuvir and ledipasvir, with or without ribavirin. 3) Sofosbuvir and ribavirin only (Fig 2). The addition of ribavirin, dose adjustments, or discontinuation of this agent was at the discretion of the treating provider. The treatment duration varied between 12 to 24 weeks based on HCV genotype, the stage of underlying liver fibrosis and prior treatment history.

### Treatment response

**Fig 3:**
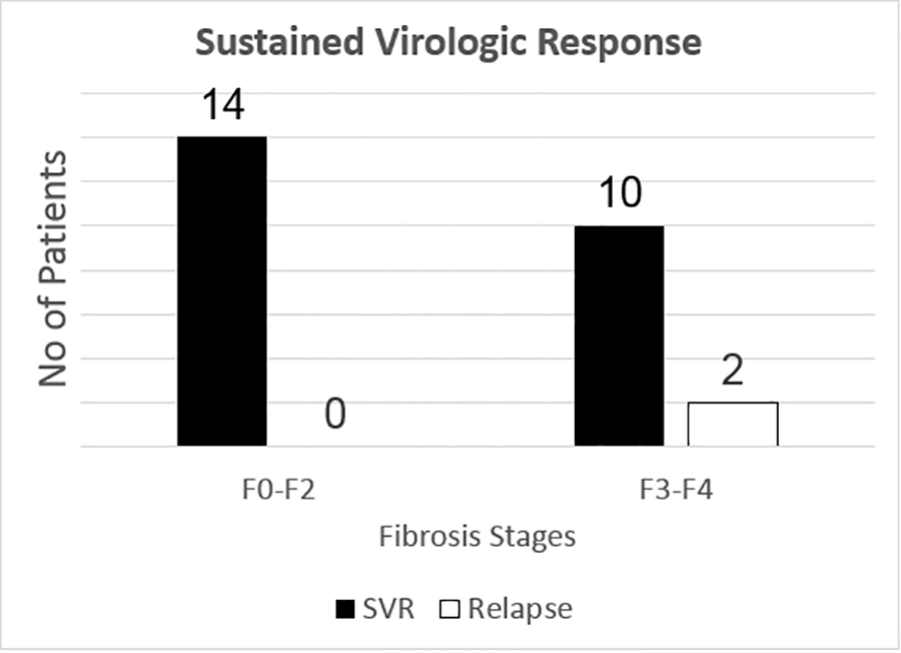
Sustained Virologic Response. Overall sustained virologic response, stratified by stage of fibrosis. The graph indicates the number of patient who achieved undetectable HCV viral load at 12 weeks post-treatment. Of note, one patient had a negative HCV viral load measured at week 4 post-treatment and was counted towards achieving SVR.

All patients completed antiviral treatment and were followed for at least 12 weeks post-treatment. There were no cases of premature discontinuations of therapy and no loss of follow-up. Twenty-three patients had SVR12 assessment; one patient had SVR4 assessment. Although based on recent publication indicating that SVR4 is an excellent predictor for SVR12, we excluded this patient from our primary endpoint analysis, hence our overall rate of viral cure (SVR12) was 91% (Fig 3).[21] On-treatment, viral suppression occurred more quickly in noncirrhotic patients compared to those with underlying cirrhosis: of the 18 patients who had available on-treatment week 4 HCV RNA, 7 out of 12 (58%) of the non-cirrhotic patients had complete viral suppression (i.e. rapid virologic response), whereas only 1 out of 6 (17%) of the cirrhotic patients had undetectable viral loads by week 4 of treatment. All patients (100%) had undetectable HCV viral load at the end of treatment. Table 2 lists the laboratory values at different treatment time points. One patient had HIV coinfection; he had HCV genotype 2 infection and was treated with sofosbuvir and ribavirin for 12 weeks. His antiretroviral regimen was emtricitabine-tenofovir and raltegravir. The HIV viral load, CD4 counts and renal function remained stable during the treatment course.

**Table 2:** Laboratory Values During and Post-Treatment.

	Baseline	Week 4	Week 12 (end of treatment)	12 weeks after treatment
HCV RNA, IU/mL, median and range	1.9 million (1060-22million)	Non-detected– 8 pts	Non-detected– 20 pts	Non-detected– 21 pts*
Cr, mg/dL, median and range	1.21 (0.66–1.76)	1.19 (0.52–2.0)	1.31 (0.43–1.8)	1.22 (0.40–1.99)
eGFR, mL/min/1.73m^2^, mean	70.9	67.4	62.2	68.4
ALT, U/L, median and range	54 (14–416)	20.5. (7–79)	18 (9–58)	15.5 (7–147)
TB, mg/dL, median and range	0.65 (0.2–8.4)	0.60 (0.2–3.5)	0.50 (0.2–1.3)	0.50 (0.2–7.6)
Hb, mg/dL, median and range	14.0 (11.6–15.2)	12.8 (8.6–13.4)	13.95 (10.1–14.7)	12.9 (10.9–15.5)

Abbreviations: Cr, creatinine; ALT, Alanine transaminase; TB, total bilirubin; Hb, hemoglobin.

* One patient achieved SVR4 but demised prior to SVR12 check point due to treatment unrelated cause. This patient was excluded from the primary end point analysis.

Two patients experienced viral relapse after completion of DAA therapy; both had advanced fibrosis or cirrhosis, with genotype 1a infection, prior HCV treatment failure, and detectable viral load at week 4 of therapy–all known risk factors for relapse. The first was a 60-year-old black male, who had received a prior combined deceased donor liver/kidney transplant from a HCV uninfected donor 3.5 years prior to initiation of DAA therapy. He had persistent genotype 1a infection, recurrent cirrhosis of the liver allograft and had previously relapsed after taking interferon and ribavirin. He was treated with sofosbuvir and simeprevir for 12 weeks. He had high baseline viral load (1.4 million IU/mL) and detectable HCV viral load at week 4 of therapy. He had negative HCV viral load at end of treatment, but relapsed by post-treatment week 4. He was subsequently re-treated with sofosbuvir, daclastavir and ribavirin for 24 weeks and he achieved SVR12. The second patient who relapsed was a 62-year-old black male, with genotype 1a infection who had undergone living unrelated kidney transplant 10 years prior to initiating DAA therapy. He had advanced liver fibrosis and was previously a non-responder to interferon and ribavirin therapy. He was treated with sofosbuvir and simeprevir for 12 weeks. He had high baseline viral load (1.2 million IU/mL) and detectable HCV viral load at week 4 of therapy. He had a negative HCV viral load at end of treatment but subsequently relapsed at post-treatment week 12. He was then re-treated with sofosbuvir and ledipasvir for 12 weeks and achieved SVR12.

### Adverse events

A total of eleven patients (46%) reported adverse events while on treatment. Table 3 lists all adverse events reported. Overall, there were 3 serious adverse events. The first patient developed atrial fibrillation in the early post-transplant period that was treated with amiodarone. He experienced pre-syncope a few days after initiation of sofosbuvir and simeprevir combination therapy and he did not seek medical attention until after 7 weeks, when he was diagnosed with sinus bradycardia with junctional escape rhythm. He continued the anti-viral therapy during the hospitalization, his amiodarone dose was decreased by half, and he had a permanent pacemaker placed. The patient has not experienced any further adverse cardiac symptoms after pacemaker placement. This event occurred approximately 10 months prior to FDA black box warning on the potential cardiac events (life-threatening symptomatic bradycardia) associated with the combination use of sofosbuvir with another DAA (in this case, simeprevir) and amiodarone. The other two serious adverse events were thought to be non-treatment related: one patient died one month after DAA treatment due to a massive gastrointestinal hemorrhage secondary to a large pseudoaneurysm from the donor aortic conduit (neoceliac axis). The other patient had partial non-occlusive portal vein thrombosis in the setting of *Streptococcus anginosus* bacteremia, possibly thrombophlebitis. The patient was treated with ceftriaxone and enoxaparin initially, and the clot subsequently completely resolved with a three-month course of warfarin. The second two serious adverse events were not felt to be related to DAA treatment. Of the seven patients that received ribavirin, two discontinued ribavirin due to side effects (fatigue, shortness of breath and gout flare), however they continued combined DAA treatment. Two of the seven patients developed anemia (defined as decrease in hemoglobin to less than 10g/dL); this improved post-treatment. When stratified by the use of ribavirin, 71% of patients who received a ribavirin containing regimen reported one or more adverse events compared to 29% of patient who did not receive ribavirin. No patients had renal transplant complications related to the treatment, including kidney rejection episodes. One patient had significant proteinuria (9 g/day) with a biopsy a month prior to treatment initiation that was indicative of vascular rejection (IIA) associated with C4d-negative glomerulitis, moderate diabetic nephropathy and no donor-specific antibodies (DSA). He was treated with methylprednisolone pulse, alemtuzumab and high-dose IVIG (2 g/kg). Four weeks later he was started on antiviral treatment (sofosbuvir/simeprevir). Proteinuria remained in the nephrotic range (5-10g/day) post-treatment despite transient reduction during treatment (S1 Fig). Repeat biopsy a year later demonstrated resolved vascular rejection but persistent C4d-negative glomerulitis, diabetic nephropathy and four glomeruli had features suggestive of collapsing glomerulopathy. There was no circulating DSA. He received further immunosuppression with methylprednisolone pulse and IVIG. He remained in complete HCV remission post-treatment with stable kidney function. Another patient with history of combined liver and kidney transplant had moderate proteinuria (1.9 g/day) of unclear etiology at treatment initiation. He was undergoing treatment with pegylated interferon and ribavirin for the previous 2 years with persistent low-grade HCV viremia. After DAA initiation, proteinuria remained around 2-3g/day and a subsequent biopsy was diagnostic of transplant glomerulopathy and focal segmental glomerulosclerosis with collapsing features without evidence of active rejection or circulating DSA (S2 Fig). He did not undergo any specific treatment and has remained on complete viral remission. His kidney function worsened slightly one year post treatment, likely related to the underlying renal pathology. One patient who received a pancreas transplant after kidney recipient who was on dual immunosuppression with tacrolimus and prednisone, underwent a 12 weeks course of sofosbuvir and ribavirin and achieved SVR12. Four months after completion of DAAs, he developed biopsy-proven pancreas allograft rejection, despite normal tacrolimus drug levels (6-8ng/mL); he was successfully treated with steroid bolus and rabbit antithymocyte globulin.

**Table 3:** Adverse Events Reported While on Treatment.

Event		Pts
Any adverse event*		11 (46%)
Any adverse event leading to discontinuation		0
Serious adverse events	Gastrointestinal bleeding	1
	Portal vein thrombosis and streptococcus bacteremia	1
	Sinus bradycardia and first degree A-V block with syncope**	1
Common adverse events	Shortness of Breath	1
	Gout flair	1
	Fatigue	1
	Headache	1
	Dizziness	1
	Diarrhea	1
	Pain in the lower extremity	1
	Photosensitivity	1
	Rash	1
	Insomnia	1

* The majority of the adverse events were clinically manageable.

**The patient who had sinus bradycardia had co-administration of sofosbuvir and amiodarone and had a pacemaker placed.

### Immunosuppression

**Fig 4:**
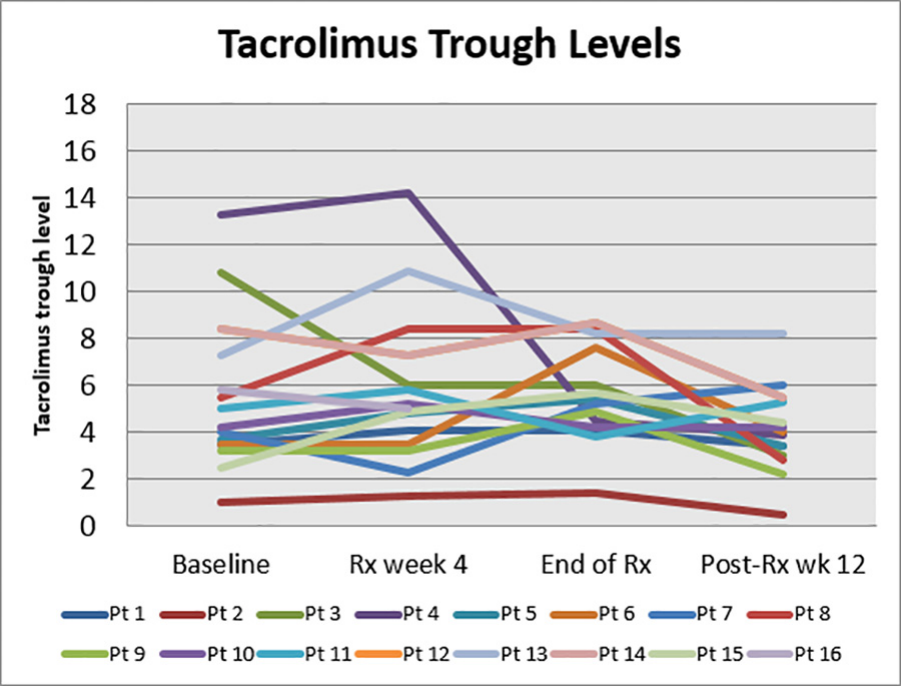
Tacrolimus Trough Levels. Tacrolimus trough levels while on antiviral treatment. The chart demonstrates the trough level of tacrolimus on the sixteen patients receiving this agent, individually, at different treatment time points.

All of the 24 patients were on at least one or more immunosuppression agents. The most commonly used drug class was calcineurin inhibitors; these were used in 22 patients (19 patients, 79%, on tacrolimus and 3 patients, 12.5%, on cyclosporine), and the most common combination therapy was tacrolimus and mycophenolate mofetil. Other agents included prednisone, sirolimus and azathioprine (Table 1). The majority of patients had stable calcineurin inhibitor trough levels during antiviral treatment with individual differences in goal trough levels according to immunological risk, single or combined organ transplant and timing after transplantation (Fig 4). One patient had a lower tacrolimus level post-treatment and one patient required dose adjustment during a hospitalization for dizziness. Only one patient was on concomitant simeprevir and cyclosporine.

### Discussion

In this study, we describe the first multicenter experience in treating patient with chronic HCV infection in the post-kidney transplant population using the all-oral interferon-free DAA regimens. The overall SVR12 rate was 91%. The two patients who relapsed post-treatment had a traditionally unfavorable treatment profile. Specifically, they were African-American, had genotype 1a infection, high pre-treatment HCV viral load, advanced underlying liver disease/cirrhosis, were previously treatment-experienced with interferon and ribavirin, and did not achieve rapid virologic response. However, both patients achieved SVR12 subsequent to re-treatment with the different DAA agents. Although our sample size was too small to detect any significant differences, the on-treatment viral kinetics were not predictive of the treatment response and the addition of ribavirin also did not appear to affect the overall SVR12 rate. The latter observation suggests that ribavirin may not be an essential component of therapy for patients with favorable treatment profiles and could potentially be omitted in patients who otherwise have relative contraindications or were intolerant. Kamar et al and Sawinski et al demonstrated in their studies a 100% SVR12 in 25 and 20 post-kidney recipients respectively.[22,23] In comparing our data, we had a more heterogeneous and complex patient population which included patients with decompensated cirrhosis, HIV coinfection and more patients with combined solid organ transplant (namely liver and pancreas). Despite this, our study demonstrated efficacy and safety in a more challenging patient population. It was also worth noting that the rapid virologic response observed in our study is lower than reported by Kamar et al, especially in the cirrhotic population. Although patient numbers are small to draw conclusion, it raises the concern that cirrhotic patients may have slower viral clearance. It was noted in Sawinski’s study that the median time for initiation of DAAs was much shorter than ours. That study did not demonstrate any significant difference in outcome when therapy was initiated within the first 6 months post-transplantation vs. later; however the patient population was small. It is possible that our cohort had a lower SVR12 rate because there was greater time for accumulation of resistance associated variants in view of the accelerated HCV viral replication that occurs due to the use of immunosuppression.

DAA therapies were well tolerated, as in previously reported series.[22,23] Adverse events were clinically manageable without DAA discontinuation. Although treatment side effects were observed to be higher in patients who received ribavirin, none led to complete treatment discontinuation; although two discontinued use of ribavirin, they completed the prescribed DAA therapy. One patient had a treatment-related serious adverse event: symptomatic bradycardia necessitating pacemaker placement. This occurred due to co-administration of sofosbuvir with amiodarone. Amiodarone is a known inhibitor of P-GP transport and sofosbuvir is partially cleared via the P-GP system.[24] A decreased in P-GP activity means patients taking amiodarone could be exposed to higher levels of sofosbuvir, which is thought to be the cause of bradycardia. This drug interaction has been described and the FDA has since issued a black box warning of drug-drug interaction between amiodarone and sofosbuvir leading to bradycardia. Only a minority of patients who were treated with ribavirin developed significant anemia; these patients did not require any blood products and their anemia reversed post-treatment. There was one death reported in our patient cohort, it occurred after DAA treatment and was not thought to be related to the treatment.

Two patients with pre-existing proteinuria were found to have evidence of collapsing glomerulopathy on kidney biopsy post-treatment. Interestingly, collapsing glomerulopathy has been reported in the context of HCV treatment with interferon use[25–27]. One of the patients was indeed exposed to interferon before treatment initiation for approximately 2 years while the other patient was not. Since significant proteinuria was present before treatment initiation, it is unlikely that the antivirals led to the collapsing feature of glomerular disease. In addition, we did not observe any significant changes in glomerular filtration rate. No patients in this cohort had known or de-novo HCV-related cryoglobulinemia.

We did not observe any significant changes in the trough level of the calcineurin inhibitors (Fig 4). Data from the liver transplant population suggest that the co-administration of simeprevir could potentially increase the serum concentration of cyclosporine and decrease the serum concentration of tacrolimus.[28–30] Only one of the patients received simeprevir and cyclosporine concomitantly and his cyclosporine trough levels were monitored closely during the treatment period and no dose adjustment was required. The most common immunosuppressive regimen used was tacrolimus and mycophenolate mofetil. One patient experienced post-treatment allograft rejection (pancreas) and we postulate that this may be related to the increased hepatic metabolism of calcineurin inhibitors as a consequence of improvement in liver function following viral eradication.[31] Overall, there were no acute kidney rejection episodes or graft loss during therapy.

There are several limitations to our study. The main limitation is the retrospective design with relatively small sample size. Thus, while serious adverse effects were rare, and the regimen was well tolerated, the number of patients in this cohort is too small to make claims about the safety of DAAs in the post-transplant period. We do not have data on the resistance associated variants or the IL28-B single nucleotide polymorphism, thus it is impossible to know if these contributed to treatment failure. We also do not have data on Q80K and as demonstrated in the OPTIMIST-2 study, the SVR12 rates were lower in genotype 1a cirrhotic patients with baseline Q80K mutation than those without the baseline Q80K mutation (74% versus 92%).[32] This could have potentially contributed to the treatment failure in our two patients. Additionally, we lacked pharmacokinetic data to monitor the drug levels at different time points as it was a retrospective study. Our study relied on routine clinical practice to follow proteinuria, mostly with urinalysis dipstick or spot urine protein/creatinine ratio in selected patients with known proteinuria; we are unable to systematically and quantitatively assess the proteinuria in all patients. While our study demonstrated efficacy, the length of follow-up was limited and future studies will be needed to determine if treatment of HCV with DAAs in the post-transplant period leads to improved long-term graft survival and overall mortality.

Our study and others suggest that HCV infection can be successfully treated after kidney transplantation. This will hopefully improve the utilization of high-risk donor organs with HCV infection and therefore reduce the waitlist time and time on hemodialysis.[13] It follows that this could translate into an overall better outcome for these patients, reduce the risk of subsequent liver disease and the need for a dual kidney-liver transplantation.

In summary, we found that the all-oral, interferon-free DAA regimens, with or without ribavirin for 12 weeks to 24 weeks, were well tolerated and highly efficacious, with an SVR12 rate of 91% among a heterogeneous and complex kidney transplant recipients population with HCV infection. Overall, only one patient experienced serious adverse events and the one reported death were not considered treatment related. The patients who relapsed had the traditional unfavorable treatment profiles, and were able to be rescued with an alternative DAA regimen. Eradication of HCV infection post-kidney transplant with DAAs is a significant advance in the care of patients with chronic HCV infection and has implications for the kidney transplant donor pool. In addition, with the recent FDA approval of elbasvir and grazoprevir for patients with chronic HCV genotype 1 and 4 with advanced renal failure will likely dramatically change the scope of the practice of HCV medicine in this patient population [33] Future studies will be needed to determine the long term effects if HCV eradication on overall graft survival and outcomes in HCV infected kidney transplant recipients.

## Conclusion

Our study demonstrated success in using an all-oral interferon-free antiviral regimen in a heterogeneous and complex post-kidney transplant population with chronic hepatitis C infection with the common genotype 1 and 2. The high efficacy and tolerability hold great promise for this patient population in improving their outcome and could enable hemodialysis patients to receive HCV positive organs, reducing their waitlist time and mortality. However, there are still many questions unanswered such as the ideal timing of treatment, liver disease severity, donor virologic status, HIV co-infection, potential drug-drug interactions and optimal choice of agents. Future and larger studies are needed to validate our findings and confirm safety.

## Supporting Information

S1 Fig Urine Protein/Creatinine Ratio for Patient 1. Urine protein/creatinine ratio of a patient with nephrotic range proteinuria upon treatment initiation. This was a 39 year-old Hispanic male with history of HCV genotype 1b, previous relapser to IFN/RBV, non-cirrhotic, with diabetes and hypertension who underwent a deceased donor kidney transplant in June 2008. Six years post-transplant (May 2014), he was found to have nephrotic-range proteinuria and a biopsy showed acute vascular rejection IIA associated with C4d-neg glomerultis, moderate diabetic nephropathy and negative donor-specific antibodies (DSA). Treatment included methylprednisolone pulse, alemtuzumab and high-dose intravenous immune globulin (IVIG) 2g/kg. Four weeks later (June 2015), he was started on HCV DAA treatment with an initial HCV viral load of 19 million IU/mL. Proteinuria remained on the nephrotic range (5-10g/day) post-treatment. Repeat biopsy a year later (May 2015) demonstrated resolved vascular rejection but persistent C4d-negative glomerulonephritis, diabetic nephropathy and four glomeruli with features suggestive of collapsing glomerulopathy. There was no circulating DSA. He received further immunosuppression with methylprednisolone and IVIG. He remained in complete viral remission post-treatment. *Abbreviations*: Bx = kidney biopsy; TP = total protein; Sof/Sim = sofosbuvir/simeviprir.
(DOCX)

S2 Fig Urine Protein/Creatinine Ratio for Patient 2. Urine protein/creatinine ratio of the second patient with proteinuria upon treatment initiation. This was a 61 year-old Caucasian male with history of HCV genotype 1a, previous relapser to IFN/RBV, cirrhotic, with diabetes and hypertension who underwent a combined deceased donor kidney and liver transplant in May 2010. He was re-treated with IFN/RBV due to liver dysfunction in 2012 for two years before switching to DAA. He had proteinuria prior to DAA treatment, around 1.8 g/day, of unclear etiology. His proteinuria remained around 2-3g/day and subsequent biopsy was diagnostic of transplant glomerulopathy and focal segmental glomerulosclerosis with collapsing features. There was no evidence of active rejection and there was no circulating DSA. He did not receive any treatment for the proteinuria and he remained in complete HCV viral remission post-DAA treatment. *Abbreviations*: Bx = kidney biopsy; TP = total protein; Sof/Sim = sofosbuvir/simeprevir
(DOCX)
