# Supplementary figures and images for: Efficacy and Safety of Direct Acting Antivirals in Kidney Transplant Recipients with Chronic Hepatitis C Virus Infection
Source: PLoS One. 2016 Jul 14;11(7):e0158431. doi: 10.1371/journal.pone.0158431 (PMC4945034; doi:10.1371/journal.pone.0158431)

Supplemental Figure 1.


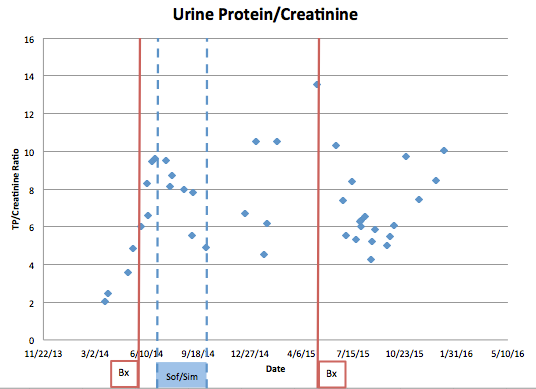

Supplement: S1 Fig — Urine protein/creatinine ratio of a patient with nephrotic range proteinuria upon treatment initiation. This was a 39 year-old Hispanic male with history of HCV genotype 1b, previous relapser to IFN/RBV, non-cirrhotic, with diabetes and hypertension who underwent a deceased donor kidney transplant in June 2008. Six years post-transplant (May 2014), he was found to have nephrotic-range proteinuria and a biopsy showed acute vascular rejection IIA associated with C4d-neg glomerultis, moderate diabetic nephropathy and negative donor-specific antibodies (DSA). Treatment included methylprednisolone pulse, alemtuzumab and high-dose intravenous immune globulin (IVIG) 2g/kg. Four weeks later (June 2015), he was started on HCV DAA treatment with an initial HCV viral load of 19 million IU/mL. Proteinuria remained on the nephrotic range (5-10g/day) post-treatment. Repeat biopsy a year later (May 2015) demonstrated resolved vascular rejection but persistent C4d-negative glomerulonephritis, diabetic nephropathy and four glomeruli with features suggestive of collapsing glomerulopathy. There was no circulating DSA. He received further immunosuppression with methylprednisolone and IVIG. He remained in complete viral remission post-treatment. Abbreviations: Bx = kidney biopsy; TP = total protein; Sof/Sim = sofosbuvir/simeviprir. (DOCX) [file pone.0158431.s001.docx]
