# Supplementary material for: Efficacy and Safety of Direct Acting Antivirals in Kidney Transplant Recipients with Chronic Hepatitis C Virus Infection
Source: PLoS One. 2016 Jul 14;11(7):e0158431. doi: 10.1371/journal.pone.0158431 (PMC4945034; doi:10.1371/journal.pone.0158431)
Supplement: S2 Fig — Urine protein/creatinine ratio of the second patient with proteinuria upon treatment initiation. This was a 61 year-old Caucasian male with history of HCV genotype 1a, previous relapser to IFN/RBV, cirrhotic, with diabetes and hypertension who underwent a combined deceased donor kidney and liver transplant in May 2010. He was re-treated with IFN/RBV due to liver dysfunction in 2012 for two years before switching to DAA. He had proteinuria prior to DAA treatment, around 1.8 g/day, of unclear etiology. His proteinuria remained around 2-3g/day and subsequent biopsy was diagnostic of transplant glomerulopathy and focal segmental glomerulosclerosis with collapsing features. There was no evidence of active rejection and there was no circulating DSA. He did not receive any treatment for the proteinuria and he remained in complete HCV viral remission post-DAA treatment. Abbreviations: Bx = kidney biopsy; TP = total protein; Sof/Sim = sofosbuvir/simeprevir (DOCX) [file pone.0158431.s002.docx]

Supplemental Figure 2
